# The Efficiency of Stem Cells (SCs) Differentiation into Functional Hepatocytes for Treating Liver Disorders: A Systematic Review

**DOI:** 10.1155/2023/4868048

**Published:** 2023-01-12

**Authors:** Marzieh Nemati, Zahra Ebrahimi, Sareh Abolhassani, Fatemeh Nemati, Bahareh Ebrahimi, Aliakbar Alizadeh

**Affiliations:** ^1^Endocrinology and Metabolism Research Center, Shiraz University of Medical Sciences, Shiraz, Iran; ^2^Department of Biology, Science and Research Branch, Islamic Azad University, Tehran, Iran; ^3^School of Dentistry, Shiraz University of Medical Science, Shiraz, Iran; ^4^Shiraz Geriatric Research Center, Shiraz University of Medical Sciences, Shiraz, Iran; ^5^Nanobiology and Nanomedicine Research Center, Shiraz University of Medical Sciences, Shiraz, Iran; ^6^Department of Tissue Engineering, School of Advanced Technology in Medicine Shiraz University of Medical Sciences, Shiraz, Iran

## Abstract

Stem cells provided new opportunity to treat various diseases, including liver disorders. Stem cells are unspecialized cells, stimulating influential research interest be indebted to their multipotent self-renewal capacity and differentiation characteristics into several specialized cell types. Many factors contribute to their differentiation into different cell types such as insulin producing cells, osteoblast, and hepatocytes. Accordingly, wide range methods and materials have been used to transform stem cells into hepatocytes, but effectiveness of differentiation is different and depends on several factors such as cell-to-cell adhesion, cell-to-cell contact, and cell biological change. Search was done in PubMed, Scopus, and WOS to evaluate results of studies about stem cells differentiation for higher efficacy. Among more than 28000 papers, 51 studies were considered eligible for more evaluations. Results indicated that most studies were performed on mesenchymal stem cells compared with other types. Acute liver failure was the most investigated liver disorder, and tissue engineering was the most investigated differentiation methods. Also, functional parameters were the most evaluated parameters in assessing differentiation efficacy. We summarize recent advances in increasing efficiency of stem cells differentiation using varied materials, since promising results of this review, further studies are needed to assess efficiency and safety of these cells transplantation in some liver disease treatment.

## 1. Introduction

There are several treatment options to cure liver diseases and among them, hepatocyte transplantation therapy is an optional cure to replace dysfunctional hepatocytes in order to significantly restore liver diseases. Still, its clinical use is limited by the lack of donor tissue. Therefore, researchers are motivated to find other treatments; in vitro differentiation of different source of stem cells (SCs) into hepatocytes or in vivo generating mature liver cells from SCs could solve this limitation [1]. SCs are unspecialized cells with a capacity of multipotent self-renewal.

Liver disorders include a wide range of both acute and chronic conditions, which are linked with remarkably morbidity and mortality across and differentiation into several specialized cell types. In vitro condition, several factors such as extracellular matrix (ECM) proteins, cell-to-cell adhesion, cell-to-cell contact, and soluble factors may affect their differentiation into specialized cell types, and play an important role in regulating the fate of stem cells [2]. Accordingly, a various range of protocols has been used to differentiate SCs toward hepatocytes. However, the efficiency of their differentiation toward functional hepatocytes, especially in response to liver disorders, are challengeable questions. Also, in vivo conditions, numerous factors are contributing to the success outcomes of cell therapy, such as the limited cell survival, cell biological changes that may occur after transplantation [3], lack of microenvironment factors, and cell integration [4]. The aim of this systematic review is to investigate the differentiation efficacy of different SC sources and various interventions toward functional hepatocytes. Assessing this review results, hampering or solving the limitation could help medical scientists to improve SCs differentiation and SCs transplantation therapy outcomes in clinical trials.

## 2. Materials and Methods

### 2.1. Focused Question

This review was conducted in accordance with the PRISMA statement to address the following question: “Which intervention has the greatest effect on the differentiation efficacy of SCs into functional hepatocytes?”

### 2.2. Search and Study Selection

Two researchers performed a comprehensive search in the PubMed and Scopus databases in June 2020. The search encompassed all types of articles using the terms included (liver cell [Title/Abstract]) OR hepatocyte [Title/Abstract]) AND (Stem Cell [Title/Abstract] OR Progenitor Cells [Title/Abstract]) OR Mother Cell [Title/Abstract]) OR Colony-Forming Unit [Title/Abstract]). Two researchers independently removed duplicates by hand screening. Inclusion criteria were the English language publications, focusing on the in vitro and animal model studies. Two researchers independently assessed all the articles by screening the title and abstracts, if necessary, the full texts were reviewed. The review articles or the SC therapy studies for diseases other than liver disorders were excluded. No data limitation regarding animal, age group, and cell type imposed. Two researchers independently inspected full texts of the potentially eligible articles. Data were collected from the full-text articles as follows: (i) the source of SCs, (ii) type of the study (in vitro or in vivo), (iii) methods used for the evaluation of SCs differentiation efficacy, and (iv) the obtained results. Efficiencies of stem cells differentiation toward functional hepatocytes were systematically reviewed and retrieved data, as the study authors had originally reported without using any specific or additional analyses were included in the manuscript. The searches were repeated in October 2022 to identify any new reports that emerged during the time to develop the manuscript and two new related articles were found and added.

## 3. Results

**Figure 1:**
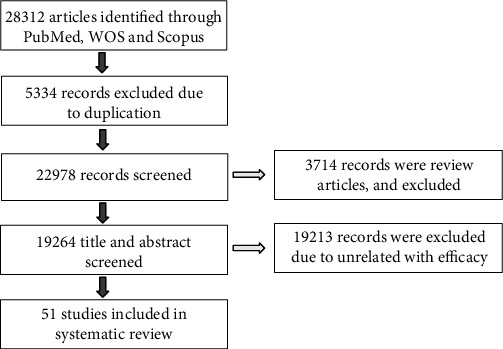
Literature search and study selection flowchart.

A total of 28312 articles were initially identified; after deleting duplications (5334 articles) by two authors using hand-screening. Among remained 22978 papers, 3714 papers were reviewed and excluded (Figure 1).

Among remained original articles (19264), 19213 were excluded due to unrelated with efficacy. By studying these articles, only 51 articles included the results of efficiency of stem cells differentiation toward functional hepatocytes.

By studying these article abstracts, only 40 articles included the results of the SC differentiation efficacy assay in vitro conditions (Table 1), and 22 papers were related to the evaluation of treatment efficacy in animal models (Table 2). Eleven studies investigated differentiation efficacy in both in vitro and in vivo condition.

Results revealed that the investigated SC cells have different sources. The SCs derived from the embryo (ESCs) were used in ten studies. Mesenchymal stem cells from various sources were employed in twenty-three studies, including bone marrow mesenchymal stem cells (BM-MSCs) in ten studies, whereas four studies investigated adipose tissue-derived mesenchymal stem cells (ADMSCs), two papers examined umbilical cord-derived mesenchymal stem cells (UCMSCs), one study investigated WJMSCs, nine articles discussed liver mesenchymal stem cells, and just two studies used hematopoietic stem cells (HSCs). Induced pluripotent stem cells (iPSCs) were used in eight studies; only one study examined the mensural stem cells and one study investigated testis-derived stem cells. By all these source variation, only five other methods were used, and these different methods had important effects on the SCs differentiation efficacy. In the following section, these studies are discussed according to the used method.

### 3.1. In Vitro Results

Application and research of different differentiation methods.

#### 3.1.1. Use of Tissue Engineering for Differentiation of SCs into Hepatocytes

Tissue engineering strategies based on the cocultivation of SCs with different cells, growth, and soluble factors [5], on the 2D and 3D scaffold [6, 7], is one of the most used methods (eighteen studies) to increase the efficacy of SCs differentiation toward functional hepatocytes.

*(1) Use of Coculture Method*. Regarding this method, different materials were used in eight studies to increase the differentiation of SCs into hepatocytes. Carraro et al. cultured human liver stem cells (HLSCs) with hepatic stellate cells, and after seven days, this in vitro condition promoted the functional differentiation of HLSCs into mature hepatocytes [5]. Chang et al. found that encapsulating MSCs with AML12 hepatocytes increased SCs differentiation toward hepatocyte-like cells, so that differentiated cells showed a 2-fold and a 2.5-fold increase in albumin (ALB) and keratin-18 (Ck-18) expression, respectively [8]. Results of another study showed that coculturing MenSCs with immortalized stellate cell line could generate functional hepatocytes and increase ALB, and CK-18 expression [9]. Another study showed that ESCs coculture with hepatic cells (HePG2) significantly increases SCs differentiation to functional hepatocytes that express ALB and Alfa Phyto protein (ALF) genes, and shows the phenotypic characteristics of liver cells [10]. Shiraki et al. demonstrated that culturing ESCs with a supportive mesoderm-derived cell line (M15) induce efficient differentiation toward functional and mature hepatocytes [11]. Results of the other investigation indicated that high number, more mature, and functional hepatocytes were generated when germ line-derived multipotent adult stem cells (MaGSCs) from mouse testis were cultured with stromal cells [12]. Coculturing BMSCs with hepatocyte growth factor (HGF) and murine fetal liver cells on laminin-coated dishes could remarkably differentiate them into hepatic-like cells expressing ALB, AFP, and CK-19 [13]. Coculturing injured hepatocytes with WJMSCs pretreated with Vitamin E reduce hepatic markers such as ALT, and mRNA level of other factors include IL-1*β* [14].

#### 3.1.2. Use of Scaffold

*(1) Scaffold Alone*. Four studies have used 2D or 3D scaffold in order to increase the differentiation of SCs toward hepatocytes. For instance, Miki et al. cultured ESCs on the hollow fiber-based 3D scaffold. Histological and functional analysis showed that this 3D scaffolds induce more functional maturation in ESCs-derived hepatocytes. This method upregulate hepatic gene expression, ammonia metabolism activity, and ALB in generated hepatocytes [6]. A study performed by Pekor et al. revealed that the fetal liver cells culture on the 3D bioreactor scaffolds could significantly induce hepatocytes differentiation [15]. Use of biodegradable and synthetic polymeric membrane promotes functional differentiation of SCs into the hepatocytes that produce ALB and urea [7]. In another study, using collagen matrix, had provide 3D scaffold and after 4 weeks had enhanced the differentiation of liver epithelial cell line (HSL) into functional hepatocyte, so that RT-PCR detected the expression of ALB, however, not detected ALB in control group [16].

*(2) Scaffold in Combination with Other Materials*. Various studies have used scaffolds in combination with other materials to increase the differentiation efficacy of SCs into hepatocytes. For instance, using a synthesized basement membrane including laminin, as a scaffold, could differentiate ESCs to functional hepatocytes expressing mature hepatocyte markers and secreting higher level of ALB [17]. Uchida et al. demonstrated that culturing amnion epithelial cells (AECs) on a 3D scaffold and treating them with isorhamnetin (a flavonoid compound) increase SCs differentiation into hepatocytes, so that in generated cells, ALB was upregulated, CYP enzyme mRNA level, ICG uptake and its release, glycogen storage activity, and urea secretion were increased [18]. Also, treating MSCs with valproic acid within the 3D collagen scaffold had significantly upregulated the expression of hepatic genes such as CK-18, AFP, and hepatic proteins such as ALB and AFP [19]. Another study showed that growing placenta MSCs on a 3D fluidized bioreactor and treating them with hepatoma-derived C3A significantly improved ALB, urea secretion, and increased CYP1A2 and CYP3A4 that indicated significant differentiation of SCs into hepatocytes [4].

#### 3.1.3. Treatment Approaches

Various combinations were used in six studies to increase the differentiation of SCs into hepatocytes. Lee et al. treated placenta-derived SCs with ginsenoside (the active compound of ginseng), then analysis morphological changes and the expression of hepatocyte-specific markers (HNF1*α*, HNF4*α* (Hepatic Nuclear Factor), and ALB) showed that ginsenoside had a greater hepatogenic differentiation of SCs than the control group [20]. The result of the other study indicated that treating SCs with Activin A, FGF-4 (Fibroblast Growth Factor), and BMP-2 [21] or with combination of HGF, Dex, and OSM, in the present or absence of insulin-like growth factor (IGF-1) [22], induced efficient hepatic differentiation. Treating different sources of MSCs (BMMSCs, or UMSCs) with HGF and oncostatin could improve the efficiency of SCs differentiation toward the hepatocytes producing ALB, storage glycogen, secretion urea, uptake LDL, and increase cytochrome P450 activity [23]. Zheng et al. found that using the inorganic calcium silicate optimized the ESCs differentiation into functional hepatocyte-like cells [24]. The other study showed that treating BMSCs with DLL4 (Delta-like ligand 4-mediated Notch activation contributes to reversing biliary injury) efficiently differentiated into Cholangiocytes and restores liver damage [25].

#### 3.1.4. Use of Extra Cellular Matrix

In 2016, Kanninen et al. had evaluated the effect of human liver progenitor HepaRG-derived acellular matrix (ACM) as a progenitor specific matrix to induce differentiation of hepatic progenitor cells into hepatocytes. RT-PCR analysis of their finding showed the higher expression of hepatic-specific markers, including CK-18, CK-19, ALB, and AFP. ACM increased further differentiation of the SCs toward hepatocytes [26].

#### 3.1.5. In Vivo Environmental Imitation

Several studies have shown that the imitation of hepatic environment, such as culturing BMMSCs with liver extraction [27] or liver tissue homogenate [28] could significantly differentiate SCs toward hepatocytes. RT-PCR data showed the higher expression of hepatic specific markers, such as ALB and AFP in experimental groups in comparison with the control group. Qihao et al. used adult liver cells to differentiate MSCs into hepatocytes. RT-PCR and immunohistochemistry analysis showed higher expression of ALB, AFP, and CK-18 in MSC and liver cells than that of control group [29]. Another study demonstrated that culturing MSCs in microfluid devices (environmental imitation) could induce hepatic differentiation efficiently. RT-PCR and immunofluorescence assays confirmed the improvement in the differentiation efficiency of MSCs toward hepatocytes, so that AFP, CK-18, HNF4, and tyrosine aminotransferase expression as well as urea production were remarkably increased [30].

#### 3.1.6. Use of Various Differentiation Protocols

Modified differentiation protocol is another method to enhance differentiation efficacy. In two studies, use of the sequentially protocol showed more noticeable differentiation of SCs into functional hepatocyte-like cells, expressing characteristic hepatocyte enzymes [31, 32]. Also, results of another study indicated that using stepwise protocol [33] or optimal culture condition [34] could efficiently differentiate SCs toward hepatic-like cells so that differentiated cells expressed higher level of hepatic specific markers such as ALB, and AFP as well as more ALB secretion [33, 34], urea production, and glycogen synthesis [33]. Application of two-step protocol decreases the differentiation time and efficiently generates highly matured and functional hepatocytes from iPSCs [35]. In addition, use of indirect coculture protocol increased the differentiation efficacy of BMSCs toward hepatocytes; upregulation of hepatic markers such as ALB and AFP, enhancement of glycogen storage, and improvement of LDL confirmed the beneficial effects of this method in SCs differentiation into hepatocytes [36]. Farzaneh et al. used hPSCs in a tank bioreactor with 150 ml working volume of physiological oxygen concentrations that led to efficient generation of functional hepatocytes, which improved liver-specific marker gene expressions [37].

### 3.2. In Vivo Results and Animal Studies

Several animal studies used different SCs or SCs-derived hepatocytes for treatment of liver disorders, then the efficacy of transplantation therapy on different animal models' liver disorders were evaluated (twenty-two studies), which the results of these studies can improve clinical investigation. Transplantation method including cell implantation (two study) and cell injection; in different sites including intravenous (2 studies) such as tail vein injection (5 studies), intraportal vein (2 studies), intrasplenic (5 studies), intrahepatic (4 studies), and intraperitoneal injections (3 studies), which will be explained and discussed the results, respectively.

#### 3.2.1. Liver Implantation

Two studies demonstrated that implantation liver MSCs-derived-hepatocytes coculturing with AML12 or PSCs, with physiological concentration of oxygen in liver damaged animals remarkably promoted liver repair, liver tissue regeneration, and reduced AST and ALT serum level [8, 37]. These results confirmed the significant SCs differentiation efficacy that could treat liver disorder and should be considered as useful methods with clinical application potential.

#### 3.2.2. Intravenous Injection

Bahmani et al. reported that intravenous injection of UMSC-CM (umbilical mesenchymal stem cells-condition media) could significantly improve liver fibrosis in male rats [38, 39]. Tail vein injection of SC-derived hepatocytes or SC-derived extracellular vesicles to animal with liver injuries had attenuated collagen deposition, liver fibrosis, and extracellular matrix alteration, it had decreased AST, ALT, increased serum ALB concentration, produced CK-18, enhanced liver regeneration and repair, and prolonged recipient survival [9, 40–42]. Portal vein transplantation of MenSCs or ADSCs to pig or female rats with liver failure or partial hepatectomy remarkably increased liver regeneration rate and upregulated the expression of hepatic markers [43, 44]. These results indicated that transplantation therapy outcomes were considerably efficient and could significantly improve liver function.

#### 3.2.3. Intrasplenic Injection

Intrasplenic or injection of SC-derived hepatocytes to human [45] or animal with liver injury or liver failure increased ALB, AFP, CK-19, and antitripcin mRNA expression, ALB secretion, serum ALB concentration, and reduced bilirubinemia. Ultimately the obtained results of these studies demonstrated the efficacy of SC-derived hepatocytes transplantation which significantly decreases liver failure [1, 21, 45–47].

#### 3.2.4. Intraliver Injection

Intraliver or intrahepatic injection of generated hepatocytes to patients [45] or animal [16, 43, 48] with liver failure or partial hepatectomy could upregulate hepatic genes expression, increase serum ALB concentration, enhance liver regeneration, and make long longevity of transplant recipient. These results are evidence of beneficial effects and success outcomes of the transplant therapy.

#### 3.2.5. Intraperitoneal Injection

Intraperitoneal transplanting of generated hepatic cells to animal with liver injury or liver deficiency led to remarkable secretion of specific liver protein such as ALB into blood circulation, reduced AST, ALT, and TB level, which showed beneficial transplantation therapy outcomes [49, 50]. Sun et al. also revealed that administration of BMSCs treated with DLL4 into immunodeficient mice with hepatic failure restored liver injury by elevating SCs differentiation toward cholangiocyte [25].

Transplanted liver epithelial cell line (HSL), cultured on collagen matrix, could significantly differentiate into hepatocyte and collagen matrix by imitating an appropriate microenvironment which increases this differentiation [16]. Transplantation of Vitamin E-WJMSCs to CCl4-induced liver injured rats after 1 month could efficiently reduce liver fibrosis [14].

## 4. Discussion

This review included the results of in vitro differentiation and animal studies. The various sources of SCs, differentiation techniques used in these studies, and the different evaluation methods highlight the advantages of comparing the obtained results with each other.

### 4.1. Cell Sources

The most cell sources used to differentiate into hepatocytes or transplantation were MSCs, especially BMSCs under variable conditions for assessing the best differentiation efficacy in liver disorders therapy.

### 4.2. Differentiation Techniques

The result of these studies indicated that the application of tissue engineering method, particularly coculturing SCs with different materials was the most used method that could significantly improve the differentiation efficacy of SCs into hepatocytes. Results of various studies have confirmed that the use of tissue engineering strategies such as coculturing method in vitro [5, 8, 13, 27–29] and in vivo [5, 45] condition and scaffold membranes [4, 6, 7, 15, 17–19] should be considered as an effective technique in SC differentiation toward hepatocytes.

Furthermore, it appears that the in vitro treatment of SCs with different factors plays a key role in the improvement of differentiation efficacy and subsequently on the therapeutic outcome. For instance, treating MSCs with plant component such as ginsenosides [20], inorganic biomaterials such as calcium silicate [24], growth factors (GFs) such as IGF [22], or GFs combination (HGF and EGF both in vitro and in vivo investigation) [51], or (HGF)/oncostatin (cytokine family member) combination [23] showed a greater differentiation of SCs into hepatocytes and would generate more functional hepatocytes. The other study showed that treating MSC with DLL4 could enhance cholangiocyte differentiation from SCs [25].

Several research groups tried to examine the effect of different extracellular matrix proteins on stem cell differentiation efficacy. The results obtained by Kanninen et al. indicated that hepatic cell differentiation by MSCs could be promoted by adding hepatic progenitor-specific matrix [26]. The use of collagen matrix by Ueno et al., as a differentiation inducer of hepatocyte from liver epithelial cell line (HSL) efficiently improved the differentiation by providing a suitable microenvironment [16].

In addition, other studies showed that using in vivo microenvironmental imitation method in in vitro differentiation condition has an effective role in increasing the effectiveness of SCs differentiation toward hepatocytes. Three studies demonstrated that restoring liver microenvironment by culturing MSCs with liver extract [27], adult liver cells [29], or with liver tissue homogenate [28] could effectively differentiate the SCs into functional hepatocytes. Another study performed by Yen et al. showed that using microfluidic device as a microenvironmental imitator could efficiently induce hepatic differentiation of MSCs [30]. The evaluation of stem cell differentiation efficacy by using different protocol has been performed in five studies. Sequential differentiation protocol by sequential addition of liver specific factors [31, 32] (in both in vitro and in vivo investigation, and in vitro condition, respectively) or culture SCs in optimal culture condition [34] showed more noticeable differentiation of SCs toward functional hepatocytes. In line with this fact, the results obtained by Song et al. and Takata et al. indicated that stepwise differentiation [33] and two-step [35] protocols could efficiently generate hepatocytes from SCs. Another study showed that indirect coculture protocol improved the differentiation efficacy of MSCs into hepatocytes [36].

### 4.3. Differentiation Protocols

#### 4.3.1. Directly or Modified Differentiation

In all in vitro investigations five modified differentiation method were used that all showed successful results in the differentiation of different source of stem cells into functional and mature hepatocytes and their difference was in the degree of efficiency, which according to the results obtained from these articles, the tissue engineering strategy has been more effective. This result may be because more articles have investigated this technique than others.

In most of the in vivo studies, transplantation has been performed as a direct stem cells therapy (undifferentiated SCs) to different sites in patient or animals with liver disorders, whose significant restore or reduce of liver disorders and results of evaluating the special liver genes and protein expression or clinical manifestation show the efficacy of differentiation SCs into hepatocytes [1, 9, 14, 21, 25, 38–44, 47–49, 52].

In the other in vivo studies differentiated [37,2,9,36,16,58,72,61] SCs were transplanted to patient or animals and exhibited liver regeneration, also, their liver function improved.

## 5. Conclusion

In summary, the results obtained from this systematic review provide better understanding about the efficacy of SC differentiation into hepatocytes for treating liver disorders. The obtained results showed that the hepatocytes derived from SCs by using tissue engineering and imitating in vivo microenvironment showed a better efficacy than the other methods of differentiation to restore liver regeneration and function. However, the obtained results indicate that more evaluation is needed about the efficacy of SC differentiation into hepatocytes for the treatment of liver disorders.

## Figures and Tables

**Table 1:** Overview of in vitro differentiation efficacy of SCs into hepatocytes.

Cell source	Intervention (s)	Outcome	Author (year)
MSCs	Cultured in biomicrofluidic cell culture device	Induced hepatic differentiation of SCs efficiently with significantly higher urea production	[30]
MSCs	Cocultured with AML12 hepatocytes	2-fold increase in ALB expression 2.5-fold increase in CK-18 expression	[8]
MSCs	Treated with valproic acid and cultured on 3D collagen scaffold	VPA-treatment cells within the 3D collagen scaffold significantly enhanced glycogen storage. Also, expression of hepatic genes: CK-18, AFP, and proteins: AFP and ALB.	[19]
BMSCs	Cultured with murine fetal liver cells (FLCs) and HGF in laminin coated dishes	Differentiated cells expressed ALB, AFP, and CK-19	[13]
BMSCs	Sequential differentiation protocol	More pronounced differentiation of SCs into functional hepatocytes	[32]
BMSCs	Cultured with liver tissue homogenate	Produced hepatocyte-like morphological characteristics, AFP expression, increasing of both urea, and ALB levels	[28]
BMSCs	Treated with DLL4	Restored damaged liver by enhancing cholangiocyte differentiation from BMSCs	[25]
BMMSCs or UMSCs	Treated with HGF and oncostatin M	Produced ALB, glycogen storage, urea secretion, uptake of LDL, and phenobarbital-inducible cytochrome P450 activity Generated functional hepatocytes	[23]
BMMSCs	Cocultured with liver cells	Strongly expressed ALB, AFP, and CK-18 in mRNA and protein level	[29]
BMMSCs	Cocultured with liver extract	Expressed hepatic specific marker genes including AFP, CK-18, and HNFa Differentiated cells were able to detoxify ammonia into urea, store glycogen, and synthesize glucose	[27]
BMMSCs	Indirect coculture system	Enhanced the differentiation efficacy of SCs, upregulation of hepatic-specific markers (ALB, and AFP), enhanced glycogen storage, and improved LDL uptake	[36]
BMMSCs	CCl4 induced liver injured mice serum	Marked increase in hepatic characteristics: High level of glycogen storage and urea production, expression of hepatic markers (ALB, CK-8, CK-18, and CK-19).	[51]
HSCs	Cocultured with injured liver cells	Expression of CK-18, HNF4, ALB, and CK-19	[42]
WJMSCs	Injured hepatocyte cocultured with WJMSCs pretreated with Vit E	Reduced ALT, ALP, and mRNA level of Cyp2e1, Hif1-*α*, and IL-1*β* to normal level	[14]
LSCs	Cocultured with hepatic stellate cells	Enhanced the functional differentiation into mature hepatic cells	[5]
PMSCs	Cultured with liver cells on 3D scaffold	Increased secretion of ALB and urea increased activity of CYP 1A2, CYP1A2, and CYP3A4,	[4]
PDSCs	Treated with Ginsenosides	Expressed several hepatocyte specific markers: HNF1*α*, HNF4*α*, and ALB Greater hepatogenic differentiation	[20]
MaGSCs	Cocultured with stromal cells	Generated high numbers of mature and functional hepatocytes	[12]
PSCs	Cultured on human liver progenitor HepaRG-derived acellular matrix (ACM)	Promoted further hepatic differentiation of PSCs into hepatocytes	[26]
hPSCs	Use of tank bioreactor with 150 ml working volume of physiological oxygen concentrations	Generated functional hepatocytes, which expressing liver-specific marker gene	[37]
MenSCs	Cocultured with stellate cells	Increasing in ALB and CK-18 expression	[9]
ASCs	Combination of HGF, Dex, and OSM in the presence or absence of IGF-I	IGF-I generated more functional hepatocyte-like cells	[22]
FLCs	Cultured on 3D scaffold	Significantly induced hepatic cells differentiation	[15]
AECs	Treated with synthesized isorhamnetin	ALB was upregulated. Increasing in CYP enzyme mRNA levels, ICG uptake and release, glycogen storage activity, and urea secretion	[18]
ESCs	Activin AFGF-4BMP-2	The differentiated cells exhibit characteristics of mature hepatocytes	[21]
ESCs	Cocultured with mesoderm-derived cell line (M15) and FGF	Could induce efficient differentiation	[11]
ESCs	Lentiviral vector, containing the fetoprotein promoter driving enhanced green fluorescent protein expression (AFP:eGFP)	Generated mature hepatocytes	[53]
ESCs	Cocultured with hepatic cells (HepG2)	Increased in the expression of liver genes: ALB, AFP, and G6P Enhanced efficient of SCs differentiation	[10]
ESCs	Stepwise cell differentiation protocol	Produced functional hepatocytes expressing: CK8, CK18, CK19, AFP, and ALB	[47]
ESCs	Membrane substratum stably expressing laminin-511	Expressing mature hepatocyte markers and secretion of a high levels of albumin	[17]
ESCs	Cultured on 3D scaffold	Upregulated hepatic gene expression, ammonia metabolism activity, ALB production, increasing in drug-induced cytochrome P450 gene expression. Induction of more functional maturation in hESC-derived hepatic cells	[6]
ESCs	Activin A, FGF-2; FGF-4, BMP2, FGF-10, HGF 4, and EGF	Produced viable cells with hepatocyte morphology, expression of alpha-antitrypsin, CK-8 and LDL receptor, producing ALB and ALT	[54]
ESCs	Cultured with calcium silicate extracts	Promote hepatic differentiation of human ESCs	[24]
ELCs	Cultured on biodegradable and polymeric membranes	Promoted expansion and functional differentiation of ELCs into hepatocytes	[7]
ESCs line (ORMES-6)	Cultured in optimal culture condition	Produced hepatocyte-like cells that expressed ALB, AFP, HNF 3, G6-P, and cytochrome P450 genes and proteins	[34]
iPSc	Stepwise protocol	Could efficiently differentiate SCs into hepatocytes	[33]
iPSCs	Two-step protocol	Efficiently generated highly matured hepatocytes	[35]
iPSCs	Cultured on matrigel	Produced functional hepatocytes	[50]
iPSCs	Hepatic differentiation media	It could efficiently generate functional hepatocytes	[55]
iPSCs	Driving of mir122 (a liver specific microRNA)	Shortened the differentiation time	[48]
PSCs	Six consecutive differentiation steps protocol	Hepatocyte-like cells that express characteristic hepatocyte enzymes	[31]

MSC: Mesenchymal stem cell, VPA: Valproic acid, CK: Cytokeratin, AFP: Alfa Phyto protein, ALB: Albumin, BMSC: Bone mesenchymal stem cell, HGF: Hepatocyte growth factor, DDL4: Delta like ligand, BMMSC: Bone marrow mesenchymal stem cell, UMSC: Umbilical mesenchymal stem cell, WJMSC: Warton Jelly mesenchymal stem cell, LDL: Low density lipoprotein, HNF: Hepatocyte nuclear factor, HSC: Hematopoietic stem cell, CCL4: Carbon tetrachloride, LSC: Liver stem cell, PMSC: Placenta mesenchymal stem cell, PDSC: Placenta-derived stem cell, MaGSC: Germ line-derived multipotent adult stem cell, MenSC: Menstrual stem cell, IGF: Insulin like growth factor, FLC: Fetal liver progenitor cell, AEC: Amnion epithelial cell, ESC: Embryonic stem cell, FGF: Fibroblast growth factor, G6P: Glucose 6 phosphate, ALT: Alanine transferase, EGF: Epidermal growth factor, ELC: Embryonic liver cell, iPSC: induced pluripotent stem cell, HSL: Liver epithelial cell line, IL-1*β*: Interleukin 1*β*, Cyp2e1: Cytochrome 450 2E1.

**Table 2:** Overview of in vivo differentiation efficacy of SCs into hepatic cells.

Cell source	Intervention (s)	Human/animals	Transplanted method	Outcome	Author (year)
MSCs	Cocultured with AML12 hepatocytes	CCl4 liver injury in rats	Implantation in liver	Significant reductions in AST and ALT levels Promoting liver repair and liver tissue regeneration	[8]
BMSCs	Transplantation	Mice with hepatic failure		Cholangiocytes can be efficiently differentiated from BMSCs	[25]
BMSCs	Cultured with HGF	Patients with end-stage liver cell failure	Intrasplenic or intrahepatic injection	Significant improvement in serum ALB The results demonstrated the short-term efficacy of SCs injection in liver cell failure.	[45]
BMMSCs	Transplantation	CCl4 liver injury in mice	Tail vein injection	Decreased AST and ALT Increased ALB concentration Improved liver regeneration	[40]
ADSCs	Transplantation	Rats with partial hepatectomy	Portal vein or hepatic parenchyma injection	Significant higher liver regeneration rate Upregulated the expression of hepatic specific markers	[43]
ADMSC	Transplantation	CCl4 liver injury in mice	Tail vein injection	Prolonged recipient survival Produced ALB and CK-18	[41]
UMSC-CM	Transplantation	CCl4 liver injury in rats	Intravenous injection	Improved oxidative status Increased expression of hepatic specific gen and protein level	[38]
UMSC-CM	Transplantation	CCl4 liver injury in rats	Intravenous injection	Improved oxidative status Increased expression of hepatic specific gen and protein level	[39]
WJMSCs	Transplantation	CCl4 liver injury in rats		Decreased liver fibrosis and fibrotic related genes	[14]
ESCs	Transplantation	Mice with liver injury	Intrasplenic injection	The resulted cells, clonally expanded hepatic progenitor cells repopulated the livers of fah-deficient mice without inducing tumorigenesis	[47]
ESCs	Transplantation	CCl4 liver injury in mice	Intrasplenic injection	Integrated into the liver and expressed *α*-1 antitrypsin	[21]
iPSCs	Transplantation	Mice with ALF	Intrahepatic injection	Prolonged recipient survival	[48]
iPSCs	Multistage differentiation protocol	Mice with liver cirrhosis	Intraperitoneal injection	Successful repopulation of the liver tissue, also secretion of human-specific liver proteins into mouse blood	[50]
iPSCs	Transplantation of differentiated cell cultured on recombinant laminin	Inherited liver disease model rats	Splenic injection	Significant bilirubinemia reduction	[46]
hPSCs	Implantation	Acute liver injury mouse		These results demonstrated the significant impact of differentiation fate and differentiation efficacy	[37]
MenSCs	Transplantation	CCL4 liver injury mice	Tail vein injection	Significant liver function improvement Attenuated collagen deposition	[9]
MenSCs	Transplantation	Pig with ALF	Portal vein injection	Significant reduction of of ALT, AST, TB, and PT levels Prolonged recipient survival	[44]
FLCs	Transplantation	Mice with ALF	Intrasplenic injection	Expressed ALB, AFP, CK-19, and antitrypsin mRNA	[1]
HSCs	Transplantation of HSCs	Noninjury sheep model of liver cell deficiency	IP transplantation	Efficiently generated functional hepatocytes secreting ALB	[49]
HSCs	Transplantation	CCL4 liver injury mice	Tail vein injection	Regenerated of injured liver by differentiating into functional hepatocytes	[42]
HSLc	Transplantation on type I collagen matrices	Analbuminemic rat	Intraliver injection	Increased in the serum ALB 2.5-fold compared to control group	[16]
Human liver SC line (HYX1)	Transplantation	Mice with ALF	Intraperitoneal	Decreased level of ALT, AST, TB, and liver injury	[52]

MSC: Mesenchymal stem cell, CCL4: Carbon tetrachloride, AST: Aspartate transferase, ALT: Alanine transferase, BMSC: Bone Mesenchymal stem cell, HGF: Hepatocyte growth factor, ALB: Albumin, BMMSC: Bone marrow mesenchymal stem cell, ADSC: Adipose derived stem cell, ADMSC: Adipose derived mesenchymal stem cell, CK: Cytokeratin, UMSC-CM: Umbilical mesenchymal stem cell-condition media, ESC: Embryonic stem cell, iPSC: induced pluripotent stem cell, ALF: Acute liver failure, MenSC: Menstrual stem cell, TB: Total bilirubin, PT: Prothrombin time, FLC: Fetal liver progenitor cell, AFP: Alfa Phyto protein, HSC: Hematopoietic stem cell, HSL: Liver epithelial cell line.

## Data Availability

The datasets used and/or analyzed during the current study are available from the corresponding author on reasonable request.
